# Does the RIFLE Classification Improve Prognostic Value of the APACHE II Score in Critically Ill Patients?

**DOI:** 10.1155/2013/406165

**Published:** 2013-08-19

**Authors:** Kátia M. Wahrhaftig, Luis C. L. Correia, Denise Matias, Carlos A. M. De Souza

**Affiliations:** Department of Postgraduate Medicine and Human Health, Bahia School of Medicine and Public Health (EBMSP), Avenida D. João VI 275, PAV.II, 2° Andar, Sala 07, Brotas, 40.290-000 Salvador, BA, Brazil

## Abstract

*Introduction.* The RIFLE classification defines three severity criteria for acute kidney injury (AKI): risk, injury, and failure. It was associated with mortality according to the gradation of AKI severity. However, it is not known if the APACHE II score, associated with the RIFLE classification, results in greater discriminatory power in relation to mortality in critical patients. *Objective.* To analyze whether the RIFLE classification adds value to the performance of APACHE II in predicting mortality in critically ill patients. *Methods.* An observational prospective cohort of 200 patients admitted to the ICU from July 2010 to July 2011. *Results.* The age of the sample was 66 (±16.7) years, 53.3% female. ICU mortality was 23.5%. The severity of AKI presented higher risk of death: class risk (RR = 1.89 CI:0.97–3.38, *P* = 0.001), grade injury (RR = 3.7 CI:1.71–8.08, *P* = 0.001), and class failure (RR = 4.79 CI:2.10–10.6, *P* = 0.001). The APACHE II had C-statistics of 0.75, 95% (CI:0.68–0.80, *P* = 0.001) and 0.80 (95% CI:0.74 to 0.86, *P* = 0.001) after being incorporated into the RIFLE classification in relation to prediction of death. In the comparison between AUROCs, *P* = 0.03. *Conclusion.* The severity of AKI, defined by the RIFLE classification, was a risk marker for mortality in critically ill patients, and improved the performance of APACHE II in predicting the mortality in this population.

## 1. Introduction 

The epidemiology, evolution, and treatment of acute kidney injury (AKI) in critically ill patients were better evaluated only after the introduction of Intensive Care Units (ICU) and the introduction of dedicated critical care medicine journals in the 1970s [1]. However, only since the 1980s, scores of disease severity were developed. 

Those scores were perfected in the 1990s, highlighting the Acute Physiology and Chronic Health Evaluation (APACHE) [2], the Therapeutic Interventions Scoring System (TISS) [3], and the Sequential Organ Failure Assessment (SOFA) [4]. The APACHE II [5] is the most often cited model in medical literature and the most used nowadays, being recommended by a ministerial order in Brazil since 1998 [6]. These prognostic models are used in the ICU to predict the outcome of patients with certain severe diseases, including acute kidney injury, and the APACHE II score has been the most commonly used predicting instrument in this population [7]. The work done that evaluated the power of APACHE II in predicting the mortality found values ranging from 0.75 to 0.90 [6, 8, 9] which were considered excellent. However, the results of the analyses of their performance in subgroups are controversial [8, 10] which have stimulated the development of specific models [11, 12]. 

The Acute Dialysis Quality Initiative (ADQI) published, in 2004, the RIFLE classification in an attempt to standardize the definition of acute renal failure [13]. The RIFLE denomination is an acronym which refers to risk (risk of renal dysfunction); injury (injury or damage to the kidney); failure (renal failure); loss (loss of kidney function); end (end stage renal disease) (Table 1). 

In several published studies in which the RIFLE classification was used, results showed a linear correlation between the RIFLE score and death, which means that the risk of death increased with the increasing severity of the disease [14–16]. Although the prognostic models have been similar to that shown by the RIFLE classification, to discriminate hospital mortality [17], this classification only takes into account the renal system, among a larger complex of disease severity. Therefore, it is likely that its performance is not better than the overall scores but may have its discriminatory values added to them. 

It is not known if the APACHE II scores, associated with the RIFLE classification, result in greater discriminatory power, in relation to mortality in critically ill patients. Thus, this study was developed to assess whether the RIFLE classification improves the performance of the overall prognostic model of disease severity (APACHE II) in those patients. 

## 2. Methods 

### 2.1. Selection of Sample

Inclusion criteria were age older than 18 years and ICU stay longer than 24 hours. The study was conducted in a tertiary hospital in Brazil, from July 2010 to July 2011. We excluded patients with a history of chronic kidney disease or kidney transplant and those who stayed less than 24 hours in the ICU. 

All patients, after being informed of the purpose of the study, signed a consent form. 

### 2.2. Study Protocol

This is a prospective observational cohort study, in which patients were followed during their ICU stay until the outcome, discharge, or death. The researcher was not a member of the patient care team and did not participate in therapeutic decisions concerning those individuals. Information about demographics, circumstances which led to hospitalization, and clinical and laboratory data was collected daily from medical records. 

The RIFLE classification was used following prerequisites for definition and classification of acute renal failure proposed by the Acute Dialysis Quality Initiative Group [14]. We did not consider the RIFLE evolutionary criteria: loss of renal function and end-stage renal function. The baseline serum creatinine (SCr) was considered as the lowest value found before admission to the ICU. When unknown, baseline serum creatinine was obtained by applying the modification of diet in renal disease (MDRD) [18] simplified formula. We considered as normal a glomerular filtration rate (GFR) of 75 mL/min/1,73 m^2^ as follows:

(1) GFR=186×−|Scr|×1.154×−|age|×0.203|0.742  if  female  |×|1.210  if  black|.

The criterion for measuring urine flow was adapted. Although all patients were using indwelling vesical catheters, allowing continuous measure and hourly recording of urine flow, only the cumulative volume was assessed within 24 hours. The weight of the patients was estimated at 60 kg assuming an average weight of well-nourished adults. Patients were classified into three categories: risk (urine flow < 30 mL/h), injury (urine flow < 18 mL/h), and, Failure (urine flow < 4 mL/h). Analyses of the criteria for diagnosis and classification of acute kidney injury were later conducted. The outcome of interest was ICU mortality. 

We defined 24-hour RIFLE as the group of patients with acute kidney injury in the first 24 hours after ICU admission and RIFLE-1 as the group of patients with acute kidney injury at any time during their ICU stay. 

For definition of sepsis and septic shock the 1991 Consensus Conference criteria settings were used [19]. 

The APACHE II [5] was calculated keeping the score for renal dysfunction, not to overestimate the RIFLE increment predictor. To avoid time-dependent bias, APACHE II was evaluated within 24 hours of admission and on the RIFLE-1 day. The most abnormal values of vital signs and laboratory exams were used. TISS scores 28 [3] and the Glasgow Coma Scale [20] (GCS) were calculated only on admission. In sedated patients GCS was recorded as the state of consciousness measured immediately before sedation. We collected all the data needed for the calculation of prognostic scores. 

## 3. Statistical Analysis

Statistical analyses were performed using SPSS version 17.0 Software (SPSS, Inc., Chicago, IL, USA). Descriptive statistics were used to characterize the population. 

Continuous variables were presented as mean ± standard deviation or as median and interquartile range, according to distribution. Categorical variables were analyzed using the *X* ^2^ test or Fisher's exact test. 

Logistic regression analysis was used to evaluate the impact of the RIFLE classification on the occurrence of mortality, adjusted for the prognosis model in question (APACHE II). 

The predictive ability of the proposed prognostic model (APACHE II score and APACHE II score incorporated into RIFLE) was assessed by the area under the curve [21] (ROC) receiver operator characteristic. To compare the ROC curves the MedCalc Software Version 12.3.0.0, Mariakerke, Belgium was used. Calibration model was performed using the Hosmer-Lemeshow [22].

## 4. Results 

The study sample consisted of 200 patients, of whom 53% were female, and mean age was 66 years (±16.7). Nonsurgical admissions were more frequent than surgical admissions (67.5% versus 32.5%), 27% with impaired respiratory tract, followed by 26% with neurological injuries and 22% due to cardiac causes. The length of ICU stay was 12 (IQR: 4–17) days. The value of the APACHE II score was 13.3 (±6.6) and 12.3 (±5.9) when scores equivalent to renal dysfunction was withdrawn. The values of TISS-28 score were 21 (±7.3) (Table 2).

The frequency of acute kidney injury within 24 hours of ICU stay was 42% classified as class risk 7%, class injury 15%, and class failure 19%. On the day of discharge or death the percentage was 47.5%. The causes most often associated with the development of acute kidney injury were septic shock 47%, sepsis 23%, and low cardiac outputs 17% and 13% of other causes. Overall mortality was 25.5%. 

**Figure 1:**
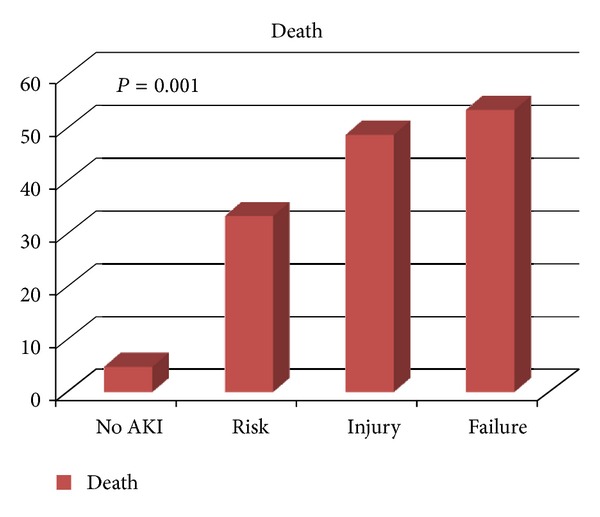
Frequency of mortality according to the RIFLE classification.

The progressive severity of AKI according to RIFLE criteria for subgroups risk, injury, and failure was associated with increased mortality when compared to patients without AKI (Figure 1). The risk of death was thus rated: class risk (RR = 1.89 95% CI: 0.97 to 3.38, *P* = 0.001); class injury (RR = 3.7 95% CI: 1.71–8.08, *P* = 0.001); class failure (RR = 4.79 95% CI 2.10 to 10.6, *P* = 0.001). 

The RIFLE classification was associated with mortality independent of APACHE II score (Table 3). 

It was observed that the APACHE II score calculated within 24 hours after admission to the ICU showed *C*-statistics of 0.73 (95% CI: 0.66 to 0.81, *P* = 0.001) in relation to prediction of death. After incorporating the RIFLE score, the APACHE II score showed statistically *C*-0.77 (95% CI: 0.70 to 0.84, *P* = 0.001). The APACHE II when calculated on the day of renal injury at any time during their ICU stay had *C*-statistics of 0.75 (95% CI: 0.68 to 0.80, *P* = 0.001) in relation to prediction of death, and after incorporating RIFLE 1 score, the APACHE II had *C*-statistics of 0.80 (95% CI: 0.74–0.86, *P* = 0.001) (Table 4). 

**Figure 2:**
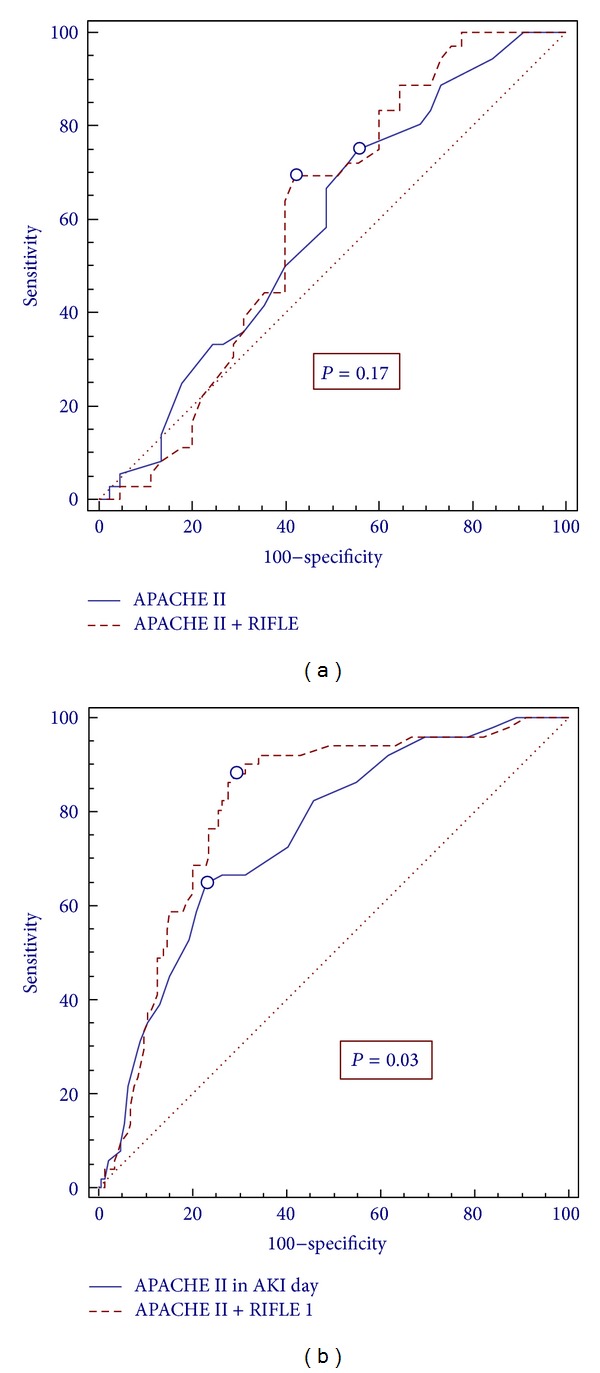
Comparative analysis between the ROC curves and the APACHE II score and calculated after incorporating the RIFLE score. (a) Defined in 24 hours after ICU admission. (b) defined at any time during ICU stay.

 Comparing the ROC curves, there was no significant difference between RIFLE and APACHE II associated with RIFLE, *P* = 0.17, when both set within 24 hours of admission, but there was significant difference between the RIFLE and Apache II associated with RIFLE, *P* = 0.03 when both were defined on the day of AKI (Figure 2). 

## 5. Discussion 

The findings of this study have demonstrated that the RIFLE score added value to the performance of APACHE II in predicting mortality. A likely explanation is that the criterion of reduction in urine flow set 73% of the cases of AKI in the most severe RIFLE score, failure. The class failure was independently associated with mortality thus by incorporating the RIFLE classification, the APACHE II included a clinical feature of acute kidney injury associated with shorter survival: oliguria. 

It was also observed that the APACHE II score showed discriminatory power in relation to ICU mortality and similarly when evaluated within 24 hours and on the day of the AKI. Conceptually, the APACHE II includes 12 physiologic variables recorded at their worst values within the first 24 hours of hospitalization. One approach that includes physiological variables without time limitation would be more accurate to predict survival chance like, for example, the value of serum creatinine, than an approach that considers those variables only during admission. 

Previous studies that evaluated the performance of APACHE II demonstrated that a prognostic model developed from the general population of patients can have controversial performance in specific subgroups, such as patients with AKI. The authors who found unsatisfactory performance of APACHE II score in discriminating mortality when compared to other prognostic models attributed such performance to timede-pendent bias, as in the study of Maccariello et al. [7]. Those authors evaluated the performance of six prognostic models in critically ill patients and the need for dialysis and found that discrimination was bad for all models. The authors calculated the scores in the 24 hours after ICU admission. 

 On the other hand, Fernandes et al. [8] compared the performance of the APACHE II score with a specific score, the ATN-ISS [11] (individual severity scores of acute tubular necrosis) in patients admitted within and outside the ICU, calculated on the day of assessment by a nephrologist, and found that in the group admitted to the ICU the APACHE II score presented *C*-statistics of 0.75, a result similar to that found in the present study. The study by Parker et al. [23] (1998) demonstrated that the best time to risk stratify patients with AKI is the use of APACHE II score on the day of dialysis, particularly when modified for the presence or absence of urinary flow (*C*-statistics: 0.74 versus 0.80, *P* = 0.005), but in that study a comparison, at different times, was not carried out. 

This study demonstrated that APACHE II score did not improve its performance significantly when incorporated into the RIFLE classification, defined in the 24 hours after ICU admission. This finding could be associated with higher mortality in the group that developed AKI during their ICU stay, compared to patients who developed AKI within 24 hours of admission (67% versus 22%). All the patients who recovered renal function, partially or totally, had developed AKI within 24 hours of admission, while the cases of developed AKI during ICU stay progressed to more severe classes. This finding confirms the idea that the development of AKI in critically ill patients is associated with disease severity. 

However, some considerations must be made: first, when the value of baseline serum creatinine is unknown, the ADQI recommends estimating its value calculated by the formula of MDRD [18], but published results on its accuracy are conflicting. Although only 20% of baseline SCr has been calculated in this study, this may have contributed to misclassification. It is known that calculated serum creatinine does not replace real creatinine, but validation of the MDRD was not the objective of this study, and second, patients are evaluated from a single research center, which requires caution when extrapolating the data presented here for other services. 

A highlight of this study is to be the first to perform a simultaneous evaluation of a general prognostic score of disease severity, APACHE II score, associated with a specific one the RIFLE score. Moreover, there are few studies that have evaluated the RIFLE score in a prospective analysis, complying with the standards recommended by ADQI. A “real-time” analysis approaches the daily reality of intensive care and provides more reliability to the data collected and statistical analyses. 

The results of this study indicate that the RIFLE classification helped to improve the performance of APACHE II in predicting mortality in critically ill patients, when set at any time during the patient's stay in ICU. They also demonstrated that the severity of kidney injury is associated with disease severity and mortality in this population. It is noteworthy that although the scores help in discussions about prognosis and risk stratification, no single model should be used to define treatments or conduct.

## Figures and Tables

**Table 1:** RIFLE classification for acute kidney injury.

Rating	GFR	Urinary output
Risk (Risk)	↑ SCr 1.5 X or **↓** GFR > 25%	<0.5 mL/kq/h for 6 h
Injury (Injury)	↑ SCr 2 X or **↓** GFR > 50%	<0.5 mL/kq/h for 12 h
Failure (Failure)	↑ SCr 3 X or >4 mg/dL or **↓** GFR > 75%	<0.3 mL/kq/h for 24 h or anuria for 24 h
Function Loss (Loss)	Total loss for 4 weeks+	
Final Stage (End)	+3 months for Dialysis	

RIFLE: Risk, Injury, Failure, Loss, End; GFR: glomerular filtration rate; SCr: serum creatinine. Adapted [13].

**Table 2:** Demographic and clinical characteristics of critically ill patients defined by the RIFLE classification.

Variable	*N* = 200
Age (years) (±DP)	66 (±16.7)
Females *N* (%)	107 (53.5)
Days of ICU stay (IQR)	12 (4–17)
Comorbidities on admission *N* (%)	
DM + SH or DLP	99 (49.5)
Cancer/Oncology Therapy	34 (17)
NYHA class IV	06 (3)
Immunossuppression	3 (1.5)
Surgical Admission *N* (%)	
ICU Admission	65 (32.5)
Compromised system *N* (%)	
Respiratory	54 (27.3)
Neurology	52 (26.3)
Heart	43 (21.7)
Polytrauma	02 (1)
Other	47 (23)
Use of Mechanical Ventilation *N* (%)	79 (39.5)
Vasoactive drugs *N* (%)	55 (27.5)
Diuretic use *N* (%)	54 (27)
Mean arterial pressure (SD)	94 (±26.7)
Serum creatinine (minimum–maximum)	1 (0.2–9.8)
APACHE II score (±SD)	13 (±6.6)
Not patched APCHE II renal (±SD)	12.3 (±5.9)
SOFA (IQR)	3 (0–5)
Nonrenal SOFA (IQR)	2 (0–4)
TISS-28 (±SD)	21 (±7.3)
Glasgow (±SD)	13.3 (±3)

RIFLE: Risk, Injury, Failure, Loss, End: DM: Diabetes Mellitus; Hypertension: hypertension DLP: dyslipidemia; NYHA class IV: heart failure functional class IV; SOFA: Sequential Organ Failure Assessment Score; Nonrenal SOFA: Sequential Organ Failure Assessment Score without the score for renal failure. APACHE II: Acute Physiology and Chronic Health Evaluation version II. Nonrenal APACHE II: APACHE II score without referring to kidney failure. TISS-28: The Therapeutic Intervention Score System; Glasgow: Glasgow Coma Scale; SD: standard deviation; IQR: Interquartile range.

**Table 3:** The Impact of the RIFLE classification criterion in ICU mortality, adjusted for APACHE II score.

Variable	OR	df	IC 95%	*P* value
APACHE II	1.66	1	1.22–2.27	0.001
RIFLE 24 h	1.07	1	1.00–1.13	0.026
APACHE II in RIFLE-1	1.04	1	0.98–1.10	0.168
RIFLE-1	2.17	1	1.50–3.14	0.001

OR: Oddis Ration (Odd Ratio); df: degrees of freedom; CI: Confidence Interval; APACHE II: Acute Physiology and Chronic Health Evaluation version II; RIFLE 24 h: RIFLE-Risk, Injury, Failure, Loss, End defined in 24 hours of ICU stay; RIFLE-1 Risk, Injury, Failure, Loss, End set on the day of AKI during their ICU stay.

**Table 4:** Comparative analysis of discrimination and calibration of APACHE II in predicting mortality in the ICU alone and when combined with the RIFLE score *calculated within 24 * hours after ICU admission and on the Day of AKI.

Score	Discrimination			Calibration	
	Area under the ROC curve	CI 95%	*PP *	GOF	*PP *
APACHE II 24 h	0.74	0.66–0.81	0.001	9.6	0.289
APACHE II + RIFLE 24 h	0.77	0.70–0.84	0.001		
APACHE II in RIFLE-1	0.75	0.68–0.80	0.001	12	0.156
APACHE II + RIFLE-1	0.80	0.74–0.86	0.001		

ROC: receiver operating characteristic; IC: confidence interval; GOF: goodness of fit; APACHE II: Acute Physiology and Chronic Health Evaluation version II; RIFLE 24 h: RIFLE-Risk, Injury, Failure, Loss, End defined within 24 hours of ICU stay; RIFLE-1 Risk, Injury, Failure, Loss, End set on the day of AKI during ICU stay.
